# Cigarette Smoke Exposure Induces Retrograde Trafficking of CFTR to the Endoplasmic Reticulum

**DOI:** 10.1038/s41598-019-49544-9

**Published:** 2019-09-20

**Authors:** Abigail J. Marklew, Waseema Patel, Patrick J. Moore, Chong D. Tan, Amanda J. Smith, M. Flori Sassano, Michael A. Gray, Robert Tarran

**Affiliations:** 1 Marsico Lung Institute, University of North Carolina, Chapel Hill, NC USA; 2 Institute for Cell and Molecular Biosciences, Newcastle University, Newcastle-upon-Tyne, UK; 3 Department of Cell Biology & Physiology, University of North Carolina, Chapel Hill, NC USA

**Keywords:** Membrane trafficking, Protein transport, Physiology

## Abstract

Chronic obstructive pulmonary disease (COPD), which is most commonly caused by cigarette smoke (CS) exposure, is the third leading cause of death worldwide. The cystic fibrosis transmembrane conductance regulator (CFTR) is an apical membrane anion channel that is widely expressed in epithelia throughout the body. In the airways, CFTR plays an important role in fluid homeostasis and helps flush mucus and inhaled pathogens/toxicants out of the lung. Inhibition of CFTR leads to mucus stasis and severe airway disease. CS exposure also inhibits CFTR, leading to the decreased anion secretion/hydration seen in COPD patients. However, the underlying mechanism is poorly understood. Here, we report that CS causes CFTR to be internalized in a clathrin/dynamin-dependent fashion. This internalization is followed by retrograde trafficking of CFTR to the endoplasmic reticulum. Although this internalization pathway has been described for bacterial toxins and cargo machinery, it has never been reported for mammalian ion channels. Furthermore, the rapid internalization of CFTR is dependent on CFTR dephosphorylation by calcineurin, a protein phosphatase that is upregulated by CS. These results provide new insights into the mechanism of CFTR internalization, and may help in the development of new therapies for CFTR correction and lung rehydration in patients with debilitating airway diseases such as COPD.

## Introduction

The cystic fibrosis transmembrane conductance regulator (CFTR) is a cAMP-activated anion channel, which resides primarily in the apical membrane of glandular and surface airway epithelia. CFTR is vital for airway surface liquid homeostasis^1^ and dysfunctional CFTR causes the autosomal recessive disease cystic fibrosis (CF)^2^. Trafficking of CFTR from the ER and Golgi apparatus to the plasma membrane is tightly regulated by a number of chaperone proteins including, but not limited to, HSP70, HSP90 and calnexin^3^. Once at the plasma membrane, CFTR is then internalized in a clathrin-dependent manner and is normally transported to early and late endosomes prior to recycling or degradation at the lysosome^4,5^. For example, deletion of F508, the most common CF mutation, causes CFTR misfolding and impaired trafficking to the plasma membrane. This in turn results in diminished anion secretion, reduced mucociliary clearance and ultimately chronic airways infection and inflammation^6^.

After formation in the ER, plasma membrane proteins usually pass through the Golgi apparatus, are trafficked to the plasma membrane and then are internalized via endosomes. From there, they are then either degraded in lysosomes or proteasomes or recycled back to the plasma membrane^7^. However, the movement of plasma membrane proteins to and from the cell surface is extremely complex^8^. For example, retrograde transport between the endosomes (early and late) and the Golgi apparatus has been well documented^9^. This phenomenon is also important for CFTR maturation and CFTR may move from endosomes to the Golgi multiple times in order to become fully glycosylated^2^. Bidirectional trafficking may also occur between the Golgi and the ER^10^. As a case in point, endosomes form contact sites with the ER to exchange cholesterol and to allow the endocytic cargo to be modified by proteins located in the plasma membrane of the ER^11^. Furthermore, in *Saccharomyces cerevisiae*, chitin synthase-III has been shown to cycle between the plasma membrane, the endosomes and the Golgi apparatus, allowing regulated expression of the enzyme. However, mammalian proteins have not yet been shown to traffic from the plasma membrane to the ER^12^.

Chronic obstructive pulmonary disease is the third leading cause of death worldwide and is primarily caused by smoking tobacco^13^. Chronic bronchitis, a phenotype of COPD, is characterized by a productive cough which lasts for two months over two consecutive years^14^. The pathogenesis of chronic bronchitis has been attributed to the formation of mucus dehydration and decreased mucociliary clearance^15^. Inhibition of CFTR by CS has been proposed as a contributing factor in the development of the chronic bronchitis form of COPD^16–19^. Indeed, we have previously observed that CS causes plasma membrane CFTR channels to be rapidly internalized in multiple cell types^16,20^. In airway epithelia, this leads to a CF-like decrease in anion secretion that contributes to dehydration of airway surface liquid. Mucus dehydration inversely correlates with the 1 sec forced expiratory volume (FEV_1_) in COPD patients^21^, suggesting that CFTR internalization and subsequent airway dehydration is relevant to COPD pathogenesis. Interestingly, CS-induced CFTR internalization is accompanied by a significant decrease in CFTR solubility, suggesting that CFTR may be aggregating after its rapid exit from the plasma membrane^16^. Despite the potential importance of this finding for both disease pathogenesis and for potential therapeutic interventions, the mechanism underlying CS-induced CFTR internalization is not well understood. Here, we have sought to determine how CS clears CFTR from the plasma membrane and to identify CFTR’s terminal intracellular location.

## Results

### Cigarette smoke-internalized CFTR dissociates internally and is taken up by clathrin-coated vesicles

**Figure 1:**
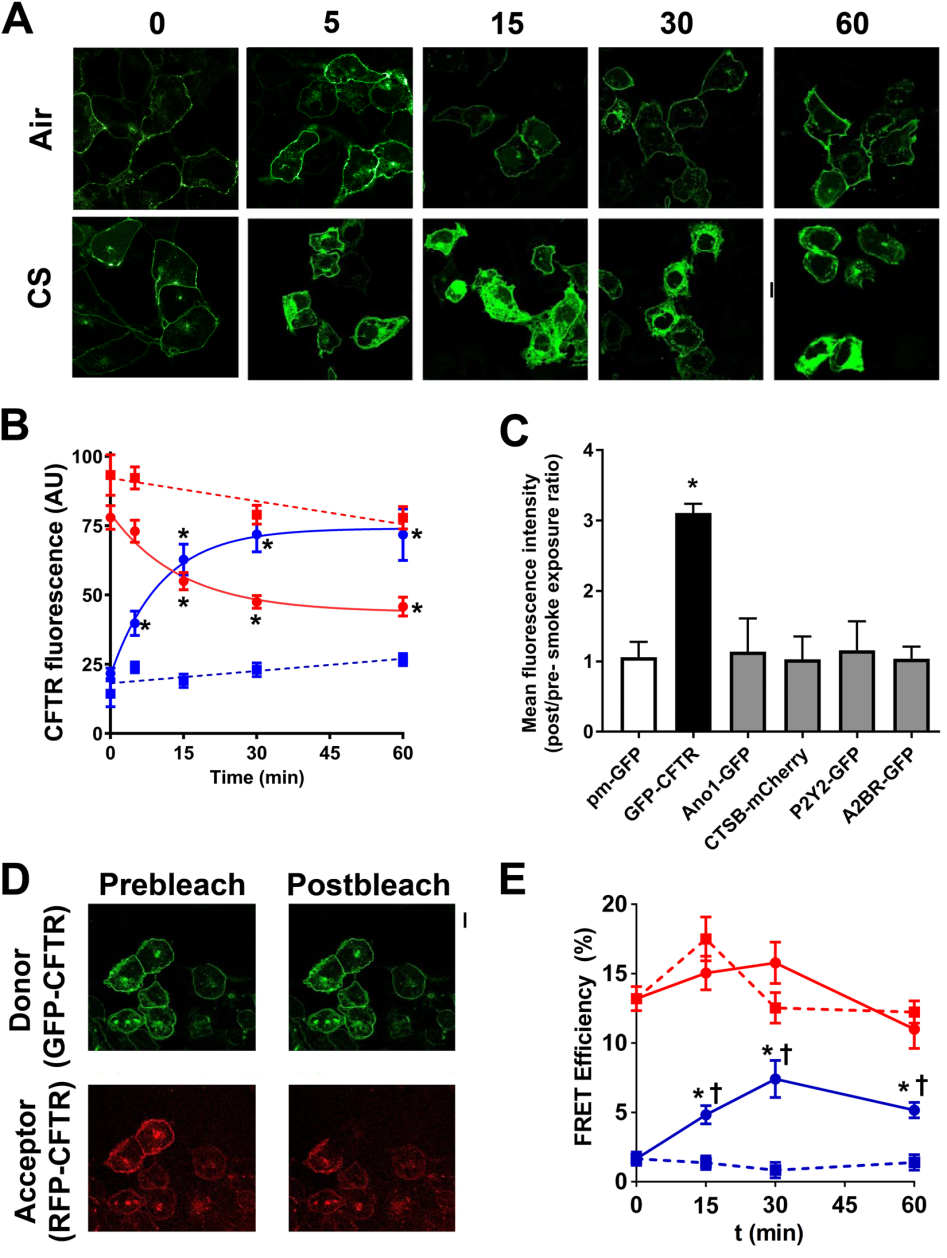
Cigarette smoke (CS) exposure causes CFTR to rapidly internalize. (**A**) Confocal micrographs showing GFP-CFTR after air or CS exposure with time. (**B**) Time course of GFP-CFTR membrane and intracellular fluorescence in HEK293T cells after air and CS exposure. CS data were fitted with single exponentials and the half-life was 10.2 min with a τ of 14.7 (n = 45–77 cells) and intracellular CFTR appeared with similar kinetics (n = 45–77 cells). Air-exposed cells were fitted with linear regression. (**C**) Bargraph showing the mean fold-change (Post-CS/Pre-CS) in intracellular fluoresence for all tested constructs. All n = 40/group from 4 separate experiments. (**D**) Confocal micrographs of GFP-CFTR (green) and RFP-CFTR (red) before and after photobleaching of the acceptor fluorophore. (**E**) Mean FRET efficiency of RFP-CFTR and GFP-CFTR measured at the plasma membrane. All n = 31–60 cells from four independent experiments. ■, plasma membrane air; ●, plasma CS; ■, intracellular air; ●, intracellular CS. *p < 0.001 different to respective air exposed cells. ^†^p < 0.001 different to respective plasma membrane control. Scale bars are 10 μm.

We have previously reported that both native and GFP-labelled CFTR are internalized after CS exposure^16,20^. GFP-CFTR matures normally and forms a fully-glycosylated band C CFTR that can internalize from the plasma membrane in a dynamin-sensitive fashion^22,23^. Here, we assessed the time course of GFP-CFTR internalization using confocal microscopy. We were unable to expose cells to CS *in situ* on the stage of the confocal microscope due to the potential for CS to damage the optics. Furthermore, given the rapid nature of CFTR endocytosis, post-CS, we were unable to expose cells to CS and image them before significant levels of endocytosis had occurred. Thus, we elected to fix cultures before, and at timed intervals after CS exposure. In HEK293T cells, air exposure did not change the subcellular localization of GFP-CFTR, whereas exposure to 13 puffs of freshly generated CS decreased CFTR membrane fluorescence intensity, with a half-life of 10.2 min and a τ of 14.7 and intracellular CFTR appeared with similar kinetics after CS exposure (Fig. 1A,B).

To see if the effects of CS were specific to CFTR or whether they extended to other plasma membrane proteins, we transfected additional, fluorescently-labelled proteins into HEK293T cells cultured in 96 well plates. We expressed GFP conjugated to a ten residue N-terminal myristoylation and palmitoylation sequence (pm-GFP), which binds to the inner leaflet of the plasma membrane^24^, the anoctamin 1 (Ano1) Cl^−^ channel, the adenosine 2B receptor (A2BR) and the P2Y2, transmembrane G-protein coupled receptors which can activate CFTR and Ano1 respectively^25^, and cathepsin B (CTSB), an intracellular and secreted protease^26^. We then used automated fluorescent microscopy to measure fluorescence before/after CS exposure. Using this approach, we found that only GFP-CFTR was internalized after CS exposure, as indicated by significant increases in intracellular fluorescence (Figs 1C; S1a,b). In contrast, PM-GFP, CTSB and the two GPCRs did not internalize (Figs 1C, S1A,B), suggesting that this phenomenon is somewhat specific for CFTR.

Using Western blotting, we have previously demonstrated that CS exposure causes a decrease in CFTR solubility in detergent, suggesting that CFTR may have aggregated^16^. Förster resonance energy transfer (FRET) can be used to measure the distance between proteins and has a resolution of ≤10 nm^27^. Therefore, we measured the FRET efficiency (%E) between GFP-CFTR (donor fluorophore) and RFP-CFTR (acceptor fluorophore) as an independent marker of aggregation. Plasma membrane CFTR FRET efficiency levels were ~15% after air exposure and CS exposure did not alter this (Fig. 1C,D). Under basal conditions, little CFTR was detected intracellularly and FRET efficiency was ~0, suggesting that normally internalized CFTR molecules are too far apart to undergo FRET. In contrast, CFTR accumulated in the perinuclear region following CS exposure (Fig. 1C,D) and FRET efficiency was ~5% post-CS exposure, which was significantly lower than plasma membrane FRET efficiency after CS exposure, but significantly higher than intracellular CFTR FRET efficiency after air exposure. Taken together, these data suggest that CFTR-CFTR interactions after CS-induced internalization were abnormal.

### Cigarette smoke induced CFTR internalization is dynamin-dependent

Hypertonic sucrose inhibits endocytosis by causing a reduction in the size and number of clathrin-coated pits^28^. Since CFTR internalizes via clathrin-coated pits^29^, HEK293T cells were pre-treated with hypertonic sucrose for 15 min before exposure to air or CS. Hypertonic sucrose had no effect on intracellular CFTR after air exposure, but significantly attenuated intracellular CFTR accumulation after CS exposure (Fig. S2A,B). HEK293T cells were then co-transfected with GFP-CFTR and clathrin light chain conjugated to mRFP. Under control (air) conditions, colocalization between GFP-CFTR and clathrin light chain-mRFP occurred (Fig. S2C,D). However, after CS exposure, the percentage of colocalization between CFTR and clathrin light chain-mRFP significantly increased for up to an hour after CS exposure, indicating that more CFTR is internalized by clathrin coated vesicles following CS exposure than during normal endocytosis (Fig. S2C,D).

**Figure 2:**
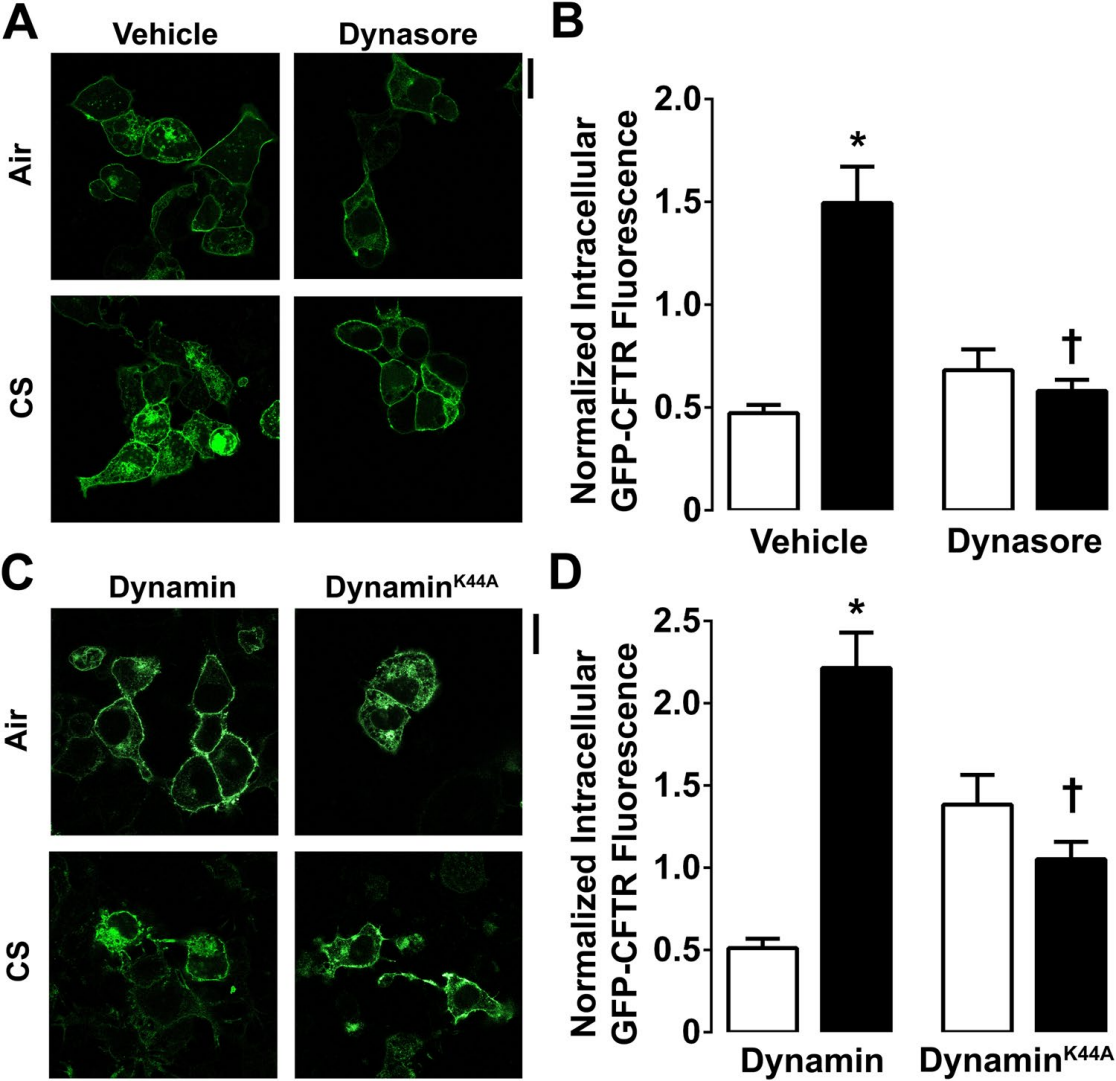
CS-induced CFTR internalization is dynamin-dependent. (**A**) Cultures were pre-treated with dynasore for 30 min at 37 °C. Representative confocal images of GFP-CFTR in the presence of vehicle or 80 µM dynasore in air and CS exposed conditions. (**B**) Mean intracellular CFTR fluorescence intensity in air (open bars) or CS (closed bars) cells treated with vehicle or dynasore (n = 101–182 cells from 3 independent experiments). (**C**) Representative confocal images of air and CS treated cells transfected with GFP-CFTR and co-transfected with wild-type dynamin or dynamin^K44A^. (**D**) Bar graphs of mean intracellular GFP-CFTR fluorescence in air (open bars) or CS (closed bars)-exposed cells transfected with CFTR and wild-type or K44A dynamin. All data points are n = 65–105 cells from 3 independent experiments. *p < 0.01 different to control, ^†^p < 0.01 different to CS control. Scale bars = 10 µm.

Dynasore is a small molecule inhibitor of dynamin that blocks dynamin-dependent internalization^30^. To further understand how CFTR is internalized after CS exposure, HEK293T cells expressing GFP-CFTR were pre-treated with vehicle or 80 µM dynasore for 30 min before CS exposure. Post-CS, intracellular CFTR fluorescence significantly increased in the presence of the vehicle and dynasore had no effect on CFTR localization after air exposure (Fig. 2A,B). However, dynasore fully prevented CS-induced CFTR internalization (Fig. 2A,B). Since dynasore has relatively low specificity and can also inhibit endocytosis in dynamin triple knockout cells^31^, we next co-expressed a dominant negative dynamin^K44A^ construct with GFP-CFTR. In the presence of dynamin^K44A^, CS-induced CFTR internalization was again inhibited (Fig. 2C,D). In contrast, wild type-dynamin had no effect on GFP-CFTR internalization, suggesting that dynamin GTPase activity is required for CS-induced CFTR internalization.

### CFTR co-localizes with early endosomes soon after CS exposure

**Figure 3:**
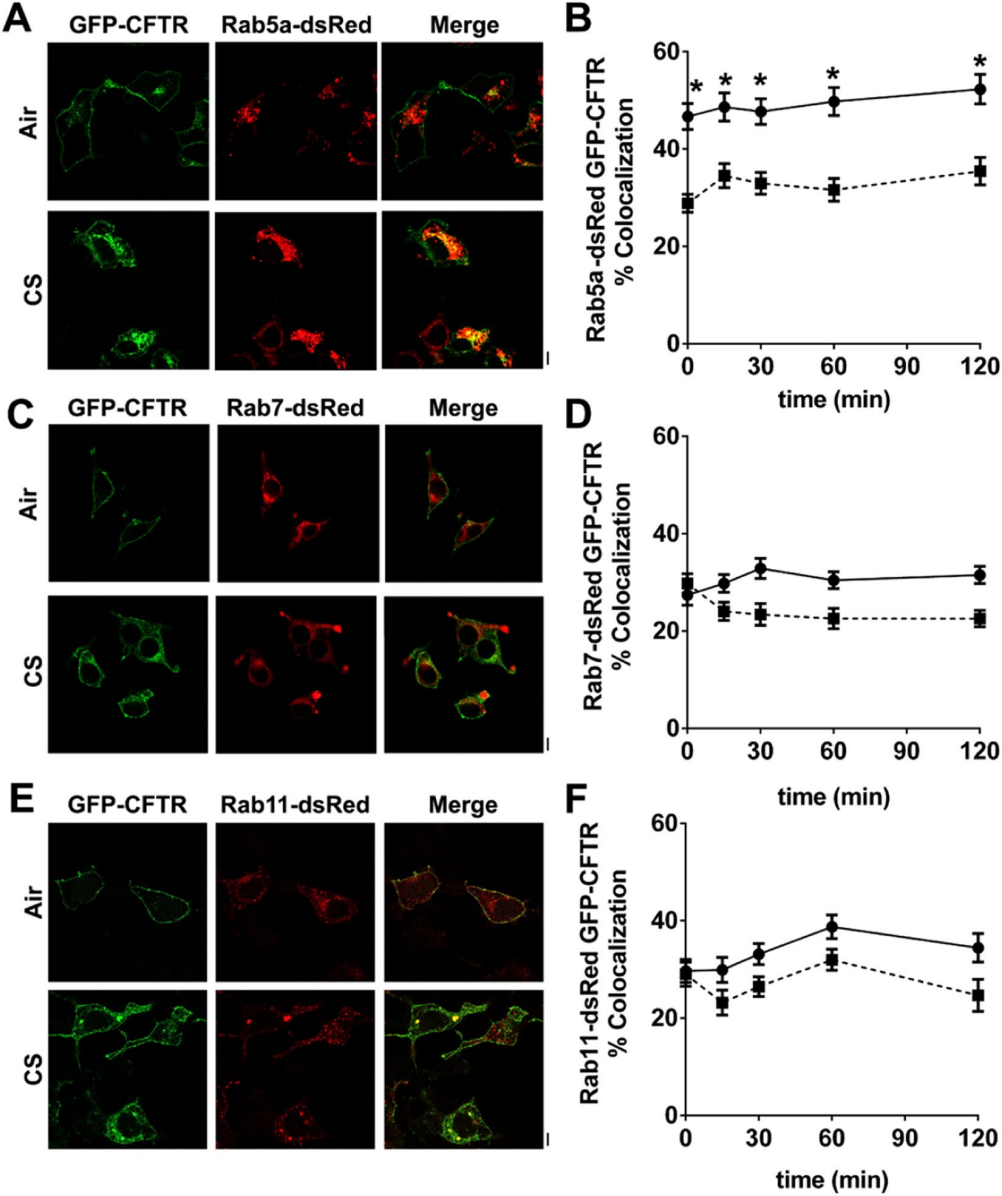
CFTR traffics through early endosomes after CS exposure. Confocal micrographs showing co-expression of GFP-CFTR with (**A**) Rab5a-DsRed, (**C**) Rab7-DsRed and (**E**) Rab11-DsRed after 15 min exposure to air or CS. (**B**,**D**,**F**) Compiled data showing mean percentage colocalization between GFP-CFTR and DsRed-tagged constructs. Each data point represents n = 25–108 cells from 3 independent experiments. ■, air exposure; ●, CS exposure. *p < 0.001 different to air controls. Scale bars = 10 µm.

After endocytosis by clathrin coated vesicles, CFTR typically traffics to early endosomes, and from there to late or recycling endosomes^29^. To determine whether CFTR still followed this pathway after CS exposure, we looked for colocalization between GFP-CFTR and Rab5A-DsRed, as a marker of early endosomes, Rab7-DsRed, a marker of late endosomes and Rab11-DsRed as a marker of recycling endosomes^32–34^. We observed basal colocalization between CFTR and Rab5A, Rab7 and Rab11 (Fig. 3A–F). However, only colocalization between CFTR and Rab5A significantly increased after CS exposure with time (Fig. 3A,B), whilst CFTR’s association with Rab7 and Rab11 was unchanged, indicating that significantly more CFTR was associated with early endosomes after CS exposure than with late or recycling endosomes (Fig. 3C,F).

### Cigarette smoke causes retrograde CFTR trafficking to the endoplasmic reticulum

**Figure 4:**
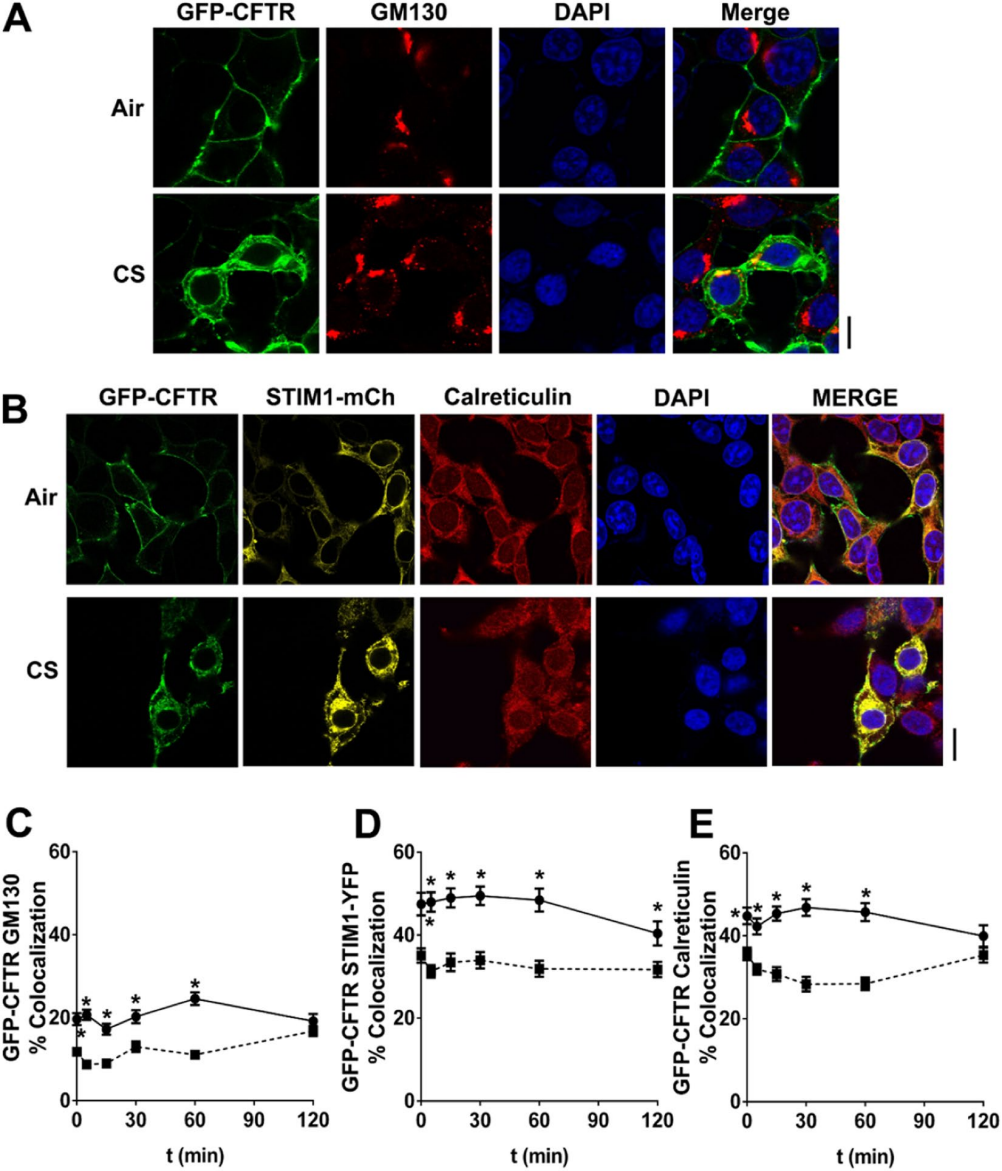
CFTR colocalizes with markers of the endoplasmic reticulum and Golgi apparatus. Confocal images of colocalization between GFP-CFTR (green) with (**A**) GM130 (antibody) and (**B**) calreticulin (antibody) and STIM1-mCherry after exposure to air or CS. DAPI (blue) was used as a counter stain. (**C**–**E**) Percentage colocalization between CFTR and GM130, STIM1-mCherry and calreticulin as indicated over time. Each time point represents n = 25–108 cells from 3 independent experiments. ■, air exposure; ●, CS exposure. *p < 0.001 different to respective air controls. Scale bars = 10 µm.

Since CFTR trafficking was markedly different after CS exposure, we used a variety of organelle markers to determine CFTR’s terminal location. To test whether GFP-CFTR entered the Golgi apparatus, an antibody specific to the *cis*-Golgi protein, GM130, was utilized. Colocalization of GFP-CFTR with GM130 significantly increased following CS-exposure compared to air controls over the initial 60 min (Fig. 4A,C). To determine whether CFTR also entered the ER after CS exposure, HEK293T cells expressing GFP-CFTR were either probed with an anti-calreticulin antibody or co-transfected with STIM1-mCherry. Post-CS exposure, both calreticulin and STIM1 displayed significantly increased colocalization with GFP-CFTR compared to air controls (Fig. 4A,D,E), suggesting that CS-induced CFTR traffics to the endoplasmic reticulum. Of note, this ER staining matched the previously observed “perinuclear” location of CFTR, which persisted for up to 24 h post CS-exposure^16^.

**Figure 5:**
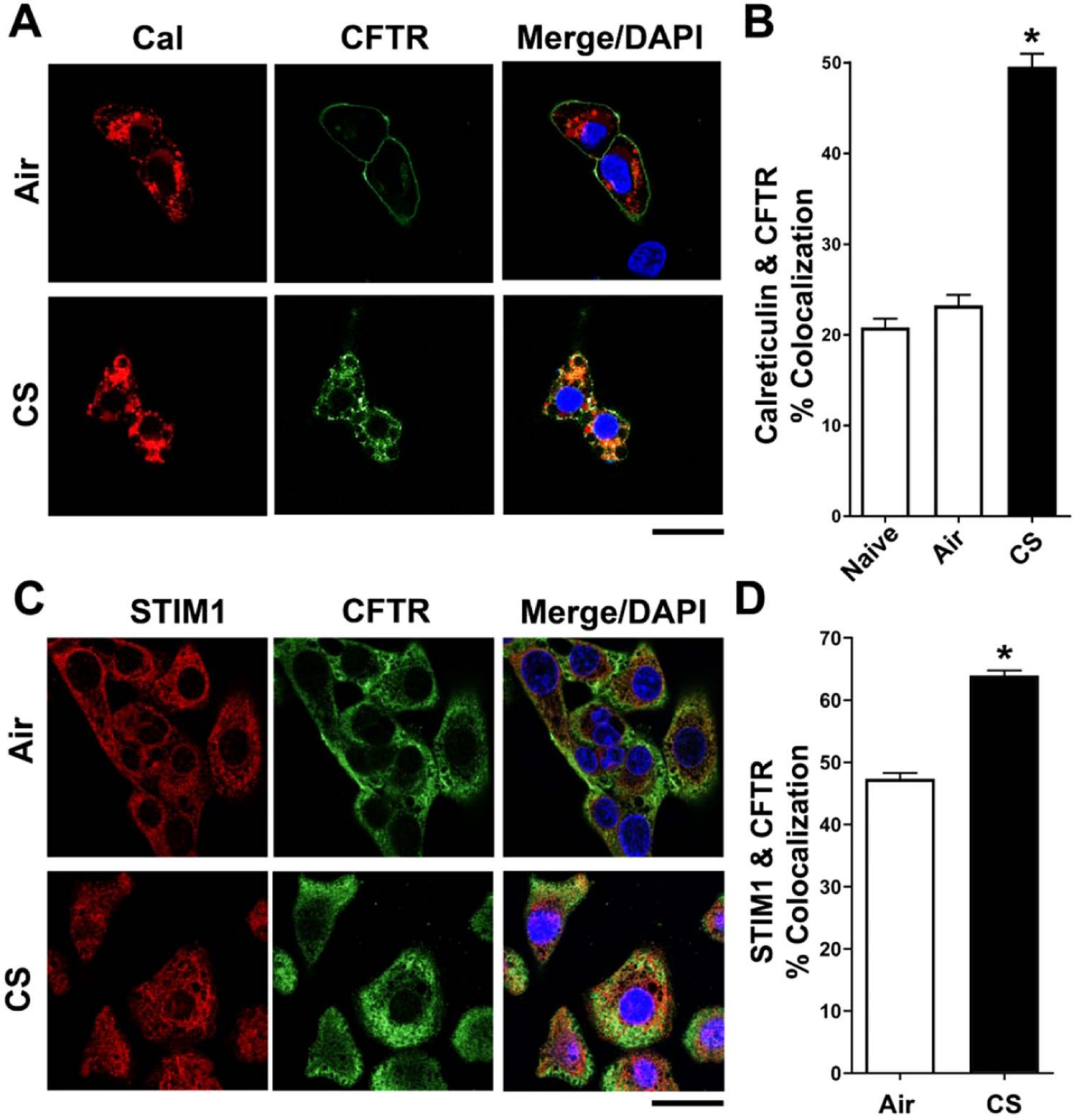
Non-GFP labelled CFTR shows increased colocalization with endoplasmic reticulum markers after CS exposure. (**A**) Representative confocal images of HEK293T cells showing calreticulin (rabbit polyclonal antibody followed by goat anti-rabbit Alexa 633 secondary, red) and a CFTR construct that has an extracellular HA epitope tag (HA-CFTR, green) followed by exposure to anti-HA mouse monoclonal primary antibody that was conjugated to Alexa-488. For these studies, HA-CFTR labeling was performed at 4 °C so as to only label surface CFTR and cells were warmed up to 37 C and air- or CS-exposed. (**B**) Bargraphs showing % colocalization between CFTR and calreticulin in naïve cells (i.e. immediate fixing with no air or smoke exposure; n = 124 cells) as well as air (n = 144 cells) and CS (n = 152 cells) exposures. (**C**) Representative confocal images of endogenous CFTR in human bronchial epithelia (probed with CFTR # 596 antibody followed by goat Alexa568 secondary, green) and endogenous STIM1 (probed with rabbit anti-STIM1 followed by goat Alexa488 secondary, red) after air or CS exposure. (**D**) Bargraphs showing % colocalization between CFTR and STIM1 after air (n = 212 cells) and CS (n = 224 cells) exposures. All experiments were performed on three separate occasions. DAPI (blue) was not used for quantification. *p < 0.001 different to control. Scale bars = 10 µm.

Next, we performed surface labelling in HEK293T cells transfected with exotope CFTR, a construct that contains a HA- tag extracellularly between the third and fourth membrane spanning domains^4^. We have previously used this construct to show that CFTR internalizes after cigarette smoke exposure^16^. Here, we chilled cells to 4 °C, blocked and exposed them to an anti-HA antibody that was directly conjugated to the Alexa488 dye so that only plasma membrane CFTR was labelled (Fig. 5A). To determine baseline CFTR levels, we then fixed some cells immediately, i.e. before they were exposed to air or CS (naïve). We then warmed the remainder of the cultures to 37 °C, exposed them to air or CS over 10 min, returned them to the to 37 °C incubator for 50 min and fixed them. Next, we blocked again, probed with the calreticulin antibody, imaged and quantified the percentage colocalization between CFTR and calreticulin. CS, but not air exposure, caused an obvious internalization of CFTR (Fig. 5A), that was accompanied by a significant increase in the degree of colocalization between CFTR and calreticulin (Fig. 5A,B). In contrast, there was no significant difference between CFTR and calreticulin colocalization after air exposure, relative to the naïve cells, indicating that post-surface labelling, the air exposure did not induce any detectable CFTR internalization.

We then searched for altered colocalization between CFTR and ER-markers in cells that endogenously expressed native CFTR. For these studies, we cultured primary human bronchial epithelia derived from normal/non-smoking donors on glass coverslips for 24 h so that we could image CFTR with a high NA objective lens (100 × 1.49 NA) in order to yield the best possible resolution. We then exposed them to air or CS (Fig. 5C). Unlike the surface labelling studies (Fig. 5A,B), this approach detected both surface and intracellular CFTR (Fig. 5C,D). However, after a 10 min CS exposure followed by a 50 min incubation at 37 °C, we found that the percentage colocalization between endogenous STIM1 and CFTR significantly increased by ~20% (Fig. 5D), indicating that the observed colocalization between CFTR and ER markers also occurred in primary airway epithelia.

### CFTR’s C-terminus and nucleotide binding domain 2 are not required for CS-induced CFTR internalization

The C-terminal domain of CFTR strongly influences CFTR turnover via several internalisation motifs^35,36^. To better understand the role of the C-terminus of CFTR in CS-induced internalization, C-terminal truncation mutants were tested. After 48 h of expression, both GFP-CFTR^L1254X^ (which lacks its PDZ motif) and GFP-CFTR^K1174X^ (which lacks the PDZ motif and nucleotide binding domain 2) still localized to the plasma membrane (Fig. S3a,b). Neither truncation prevented CS-induced CFTR-internalization, and the intracellular accumulation of these mutants was not different to that of wild-type GFP-CFTR (Fig. S3a,b), indicating that CFTR’s C-terminus was not involved in CS-induced internalization.

### Cigarette smoke causes dephosphorylation of CFTR leading to its internalization

**Figure 6:**
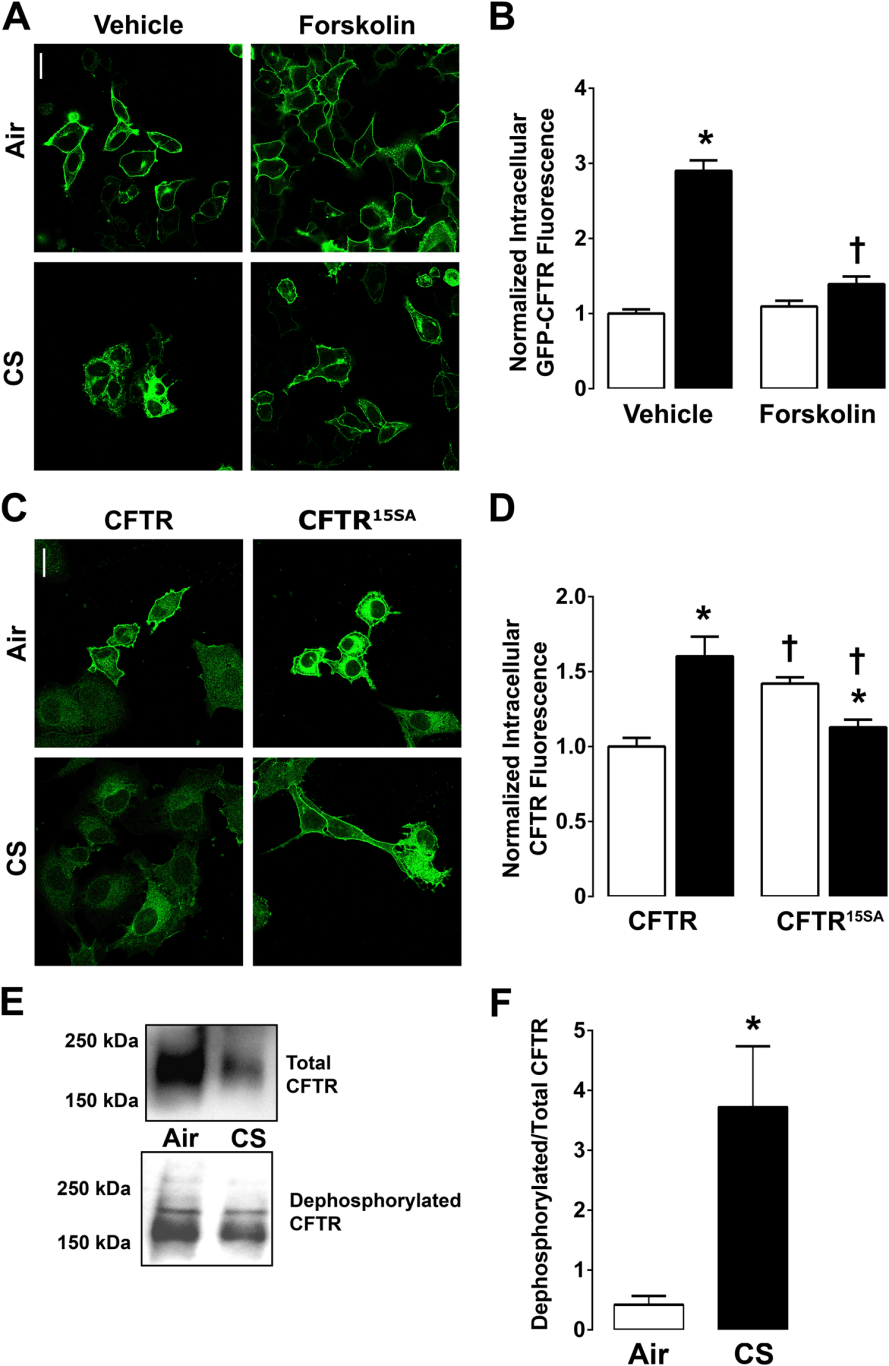
CS-induced CFTR dephosphorylation is required for CFTR internalization. (**A**) Representative confocal micrographs of GFP-CFTR in the presence of vehicle or 5 µM forskolin follwed by air or CS exposure. (**B**) Bargraph of mean intracellular CFTR fluorescence after air (open bars) or CS (closed bars) with vehicle or forskolin. N = 137–192 cells from 3 independent experiments. (**C**) Images of BHK cells stably expressing either CFTR or CFTR^15SA^ exposed to air or CS. Cells were fixed, permeablized and labelled with anti-CFTR 596 antibody and secondary anti-mouse antibody conjugated to alexa 488. (**D**) Mean intracellular CFTR fluorescence in air (open bars) or CS (closed bars) exposed cells transfected with wild-type or CFTR^15SA^. N = 80–236 cells from 3 independent experiments. (**E**) Typical western blots followed by apical surface biotinylation. Blots were probed for total and dephosphorylated CFTR and mean densiotometry of dephosphorylated**/**total CFTR is shown in (**F**). Gel blots were cropped from original gel images included in the Supplementary Information section (Figure S4). Air (open bars); CS (closed bars). 3–5 cultures from 3 individual experiments. *p < 0.05 different to air controls. ^†^p < 0.01 different to respective air or CS control. Scale bars are 10 µm.

Given that known motifs for CFTR endocytosis were not required for CS-induced CFTR internalization, we assessed the role of other domains in this process. Forskolin, an adenylyl cyclase agonist, stimulates cAMP/PKA-dependent phosphorylation of CFTR’s regulatory R-domain^37^. Following air exposure, forskolin did not alter CFTR’s localization (Fig. 6A,B). However, pre-treatment with forskolin significantly attenuated CS-induced GFP-CFTR internalization (Fig. 6A,B). We have previously shown that CFTR is internalized after CS exposure in multiple cell types including airway epithelia, HEK293T cells and BHK cells^16,20^. To further establish the role of PKA phosphorylation in CS-induced CFTR internalization, BHK cells stably expressing CFTR with 15 serines replaced with alanines (CFTR^15SA^) were utilized^38^. This construct lacks all predicted serine phosphorylation sites and has previously been shown to be markedly resistant to PKA-dependent R-domain phosphorylation^38^. The behaviour of CFTR^15SA^, was consistent with our hypothesis that CFTR must be phosphorylated in order to remain in the plasma membrane: Indeed, more CFTR^15SA^ failed to traffic to the plasma membrane under basal conditions and was evident intracellularly (Fig. 6C,D), and the localization of CFTR^15SA^ resembled that of CS-exposed wild-type CFTR. Interestingly, after CS exposure, the amount of intracellular CFTR^15SA^ was moderately decreased, suggesting that this construct may have been degraded. To further understand this phenomenon, we probed CFTR’s phosphorylation status in HBECs after CS exposure. We used CFTR antibody 596, which is directed against nucleotide binding domain 2 to determine total CFTR levels, and antibody 217 which is directed against the R-domain and only binds to dephosphorylated CFTR^38^ (Fig. 6E,F; the original gels are shown in Fig. S4). Importantly, our data demonstrated that CS exposure dephosphorylated CFTR, suggesting that CFTR phosphorylation is required for CFTR plasma membrane stability.

### Cigarette smoke-induced CFTR internalization is mediated by calcineurin

**Figure 7:**
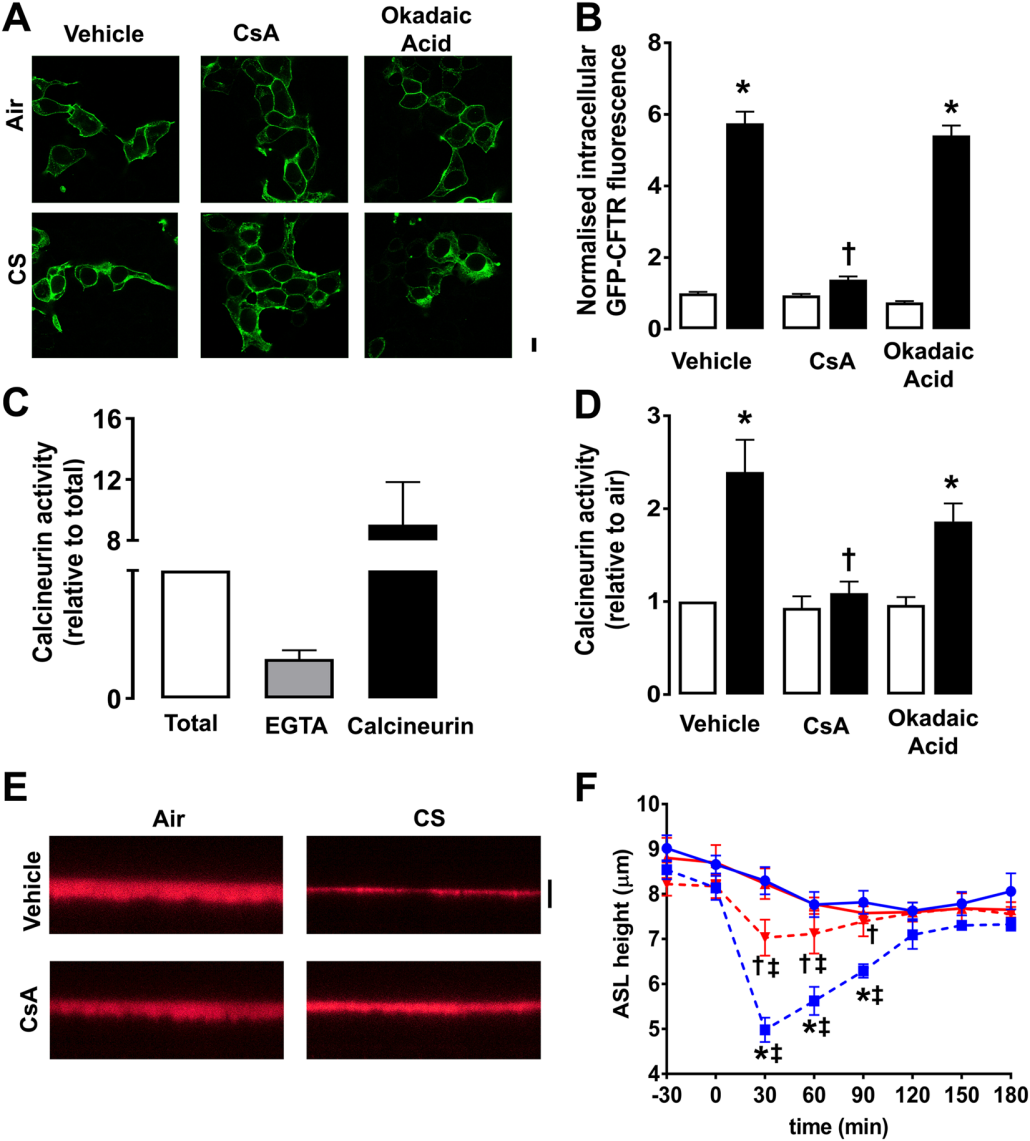
Activation of calcineurin is required for CS-induced CFTR internalization. (**A**) Representative confocal microgrpahs showing the effect of pre-treatment with vehicle, 1 µM cyclosporin A (CsA) or 10 nM okadaic acid on GFP-CFTR localization in HEK293T cells exposed to air or CS. (**B**) Bargraph showing mean intracellular GFP-CFTR fluorescence after exposure to air (open bars) or CS (closed bars) with treatment as indicated. N = 235–300 cells from 3 independent experiments. (**C**) Changes in calcineurin phosphatase activity were measured by ELISA after pre-treatment with EGTA (gray bars) or recombinant calcineurin (closed bars). Data have been normalized to total phosphatase activity. (**D**) Changes in calcineurin activity under conditions employed in (**A**). Data have been normalized to air exposed samples (n = 7 cultures per condition from 4 separate experiments) Air exposure (open bars); CS exposure (closed bars). (**E**) Typical XZ confocal images of airway surface liquid (ASL) height labelled with tetramethylrhodamine-dextran in HBECs pretreated with vehicle or cyclosporin A and then exposed to air or CS. Images were taken 30 min post- air or CS exposure. (**F**) Time course showing changes in ASL height under the conditions indicated (n = 7–8 cultures per time point from 3 donors). ●, air + vehicle; ▲air + CsA; ■ CS + vehicle; ▼, CS + CsA. *p < 0.01 different to air; ^†^p < 0.05 different to CS; ^‡^p < 0.05 different to t = 0. Scale bars are 10 µm.

Since we determined that CS causes CFTR dephosphorylation, we next considered which phosphatases were responsible for this phenomenon. Protein phosphatase 2A (PP2A) has previously been shown to regulate CFTR phosphorylation levels^39^. Okadaic acid, an inhibitor of PP2A, had no effect on CFTR’s cellular location (Fig. 7A,B). However, cyclosporin A, an inhibitor of calcineurin (PP2B), attenuated the intracellular accumulation of CFTR compared to vehicle control after CS exposure (Fig. 7A,B). We then used an ELISA assay to test whether calcineurin was activated by CS. As controls, we demonstrated that EGTA decreased calcineurin activity and that recombinant calcineurin was active (Fig. 7C). Importantly, increased calcineurin activity was detected following CS but not air exposure and this activity was attenuated by cyclosporin A but not by okadaic acid (Fig. 7D). To determine whether the effects of calcineurin were functionally significant, we measured CFTR-mediated fluid secretion in HBECs (Fig. 7E,F). As previously described^16^, CS rapidly decreased airway surface liquid height within ~30 min of exposure (Fig. 7E,F). This reduction in airway surface liquid height was prevented by cyclosporin A pretreatment (Fig. 7E,F). These data indicate that dephosphorylation of CFTR by calcineurin is required for CS induced inhibition of CFTR activity.

## Discussion

CS-induced CFTR dysfunction and subsequent airway dehydration have been previously described^16–19^. However, the underlying etiology is poorly understood, so here, we set out to better understand this phenomenon. We observed that CFTR was cleared from the plasma membrane following CS exposure with a half-life of ~10 min (Fig. 1A,B). Consistent with our previous study^20^, we did not detect changes in Ano1 localization (Figs 1C, S1). Similarly, we did not detect changes in the A2B adenosine receptor, a GPCR that is known to interact with CFTR^25^ or other proteins, including the P2Y2 receptor, or pm-GFP that bound to the plasma membrane and served as an additional control (Figs 1, S1). It has previously been suggested that CFTR gene expression is affected by CS exposure^40^. However, given the rapid onset of CFTR internalization (Fig. 1A,B), it is unlikely that the change in CFTR localizaiton was due to altered gene expression. Importantly, this time course served as a guide for our subsequent FRET and colocalization studies. Whether or not CFTR is a monomer or a dimer is controversial^41,42^. However, we detected plasma membrane FRET between GFP-CFTR and RFP-CFTR under control conditions, which may indicate dimerization, or may be due to CFTR’s membership of a larger, macromolecular complex^43^. CFTR’s solubility decreases after CS exposure^16^ and here we found that intracellular FRET efficiency was significantly greater after CS exposure than for air controls, suggesting abnormal CFTR aggregation/trafficking.

Pre-treatment of HEK293T cells expressing GFP-CFTR with hypertonic sucrose or dynasore had no effect on CFTR’s cellular distribution under control conditions, but abolished CS-induced CFTR internalization (Figs S2A,B; 2a,b), thus demonstrating that clathrin and dynamin are necessary for this effect. However, co-expression of a dynamin dominant negative construct (dynamin^K44A^) lead to a basal internalization of CFTR and also abolished further CS-induced changes in CFTR endocytosis. Taken together, these data indicate that CFTR internalizes in a clathrin/dynamin-dependent fashion^22^. However, since dynamin^K44A^ may also have affected basal CFTR localization, there may be wider implications for dynamin in channel trafficking. For example, expression of dynamin^K44A^ also increases big conductance K^+^ channel activity^44^. However, further studies will be needed to fully appreciate the role of dynamin in basal CFTR localization. Colocalization between CFTR and clathrin light chain significantly increased following CS exposure for up to an hour (Fig. S2C,D). However, this association waned over time, suggesting that CFTR was no longer being internalized beyond 1 h of CS exposure. These data indicated that the first step in removal of CFTR from the plasma membrane following CS exposure is conventional and is initiated soon after CS exposure, but is not persistent. Alpha 1 anti-trypsin, a secreted protease inhibitor is also internalized in a clathrin-dependent fashion. However, alpha 1 anti-trypsin uptake is attenuated by cigarette smoke extract exposure^45^. Furthermore, phagocytosis by macrophages, which is a modified form of endocytosis, is also attenuated following smoke exposure^46^. Thus, we hypothesize that clathrin-mediated endocytosis is normal after CS exposure and that altered internalization is protein-specific.

Internalized CFTR normally passes through early and late endosomes and then either returns to the plasma membrane via recycling endosomes or is degraded in lysosomes^4,5^. After CS exposure, CFTR levels significantly increased in the early, but not late, or recycling endosomes (Fig. 3). Consistent with the rapid clearance of CFTR from the plasma membrane (Fig. 1), some of these changes, e.g. association with clathrin, were quite rapid and occurred within 5 min (Fig. 2), i.e. before CFTR internalization reached a steady state (~30 min, Fig. 1). These data may indicate that CFTR maturation from early to late endosomes is disrupted by CS. Alternatively, endosomal function may be normal and CFTR trafficking may be abnormal after CS exposure. Further experimentation will be required to differentiate between these two possibilities. However, given that few proteins other than CFTR have been reported to be internalized after CS exposure, it is likely that CFTR internalization, not endosomal maturation, is affected following CS exposure. Whilst we did not detect CFTR entering recycling endosomes after CS exposure, we observed increased colocalization between CFTR and the cis-Golgi marker GM130 post-CS, which peaked at 60 min and waned beyond this time point (Fig. 4). In mammalian cells, enzymes and cargo proteins such as furin and the mannose-6-phosphate receptor are shuttled bi-directionally between early and late endosomes and the Golgi apparatus^47,48^. Indeed, the trans-Golgi network receives approximately 5% of its glycoproteins from the plasma membrane^49^. Not only does retrograde trafficking occur between the plasma membrane, the endosomes and the Golgi, bidirectional trafficking between the Golgi and the ER is well documented, and is thought to be dependent on COPI and COPII vesicles^10^. Since CFTR is not increased in late or recycling endosomes after CS exposure, we surmise that CFTR routes from early endosomes to the Golgi after CS exposure.

Endosomes form contact sites with the ER, which increases as the endosome matures^11^, suggesting that these organelles can directly interact. The ER-resident protein STIM1 aggregates to the ER-plasma membrane junction and is in contact with plasma membrane proteins, and direct contacts exist between the plasma membrane and the ER^50^. However, despite the documented contact sites between endosomes, the plasma membrane and the ER, the transfer of mammalian proteins from the plasma membrane to the ER is less common. We have previously shown that CS-internalized CFTR does not reach lysosomes and instead has a perinuclear location^16^. GFP-CFTR also became perinuclear within ~10 min post-CS exposure (Fig. 1A,B). CS increased CFTR’s colocalization with two different ER markers (STIM1 and calreticulin), which persisted after association with both early endosomes and the Golgi apparatus had waned. This suggested (i) that CFTR’s perinuclear location is the ER and (ii) that this is CFTR’s terminal location after CS exposure (Fig. 4B,C). This internalization from the plasma membrane and subsequent increase in colocalization with ER markers was observed not only with GFP-CFTR, but also upon performing pulse chase-type experiments with HA-tagged CFTR (Fig. 5A,B) and with endogenous CFTR expressed in airway epithelia (Fig. 5C,D). Importantly, our data also indicate that this transition occurs rapidly, since co-localization occurred within 5 min post-CS exposure (Fig. 4). Whether or not CFTR can move directly from the plasma membrane to the ER via plasma membrane ER junctions, or whether it must first pass through early endosomes and/or the Golgi apparatus is not known. There is also the perplexing question of why CFTR is shuttled to the ER. CS exposure triggers an unfolded protein response in the ER^51^. Thus, we speculate that an accumulation of CFTR in the ER may facilitate the unfolded protein response and the cellular adaptation to the stress of CS exposure.

CFTR mutants lacking the C-terminal motifs required for normal endocytosis (K1174X and L1254X) still internalized after CS exposure, indicating that CS-induced internalization was not mediated by altered PDZ-binding (Fig. S3). However, whilst the C-terminus of CFTR does not appear to be important in CS-induced CFTR trafficking, this region of CFTR can modulate CFTR surface density. For example, the CFTR-associated ligand (CAL) is a Golgi-associated protein that possess a PDZ domain that can bind to CFTR’s C-terminus, causing increased cellular retention and decreased plasma membrane CFTR^52^. The C-terminus can also interact with other proteins including microtubule-associated serine/threonine kinase 205 (MAST205), which can regulate CFTR expression and can compete with CAL for binding^53^. Inhibition of CAL can increase CFTR trafficking and has been proposed as a therapy for CF^54^. However, it would be interesting to see if CAL inhibition could also reverse CS-induced CFTR internalization. Indeed, this may be a novel therapeutic approach for treating CFTR dysfunction in COPD patients.

The cAMP-activated protein kinase A (PKA) extensively phosphorylates CFTR’s R-domain, which reduces endocytosis^55,56^. Forskolin activates adenylate cyclase to raise cAMP and activate PKA. We found that forskolin prevented CS-induced internalization of CFTR (Fig. 6A,B). Thus, to further investigate the effects of phosphorylation on CS-induced CFTR internalization, we used a PKA-unresponsive CFTR where 15 consensus sites for PKA phosphorylation, primarily in CFTR’s R-domain, were mutated to alanines (CFTR^15SA^). Billet *et al*. demonstrated that plasma membrane levels of surface biotinylated CFTR^15SA^ were similar to wild-type CFTR^57^. However, we observed that there was an intracellular accumulation of CFTR^15SA^, even under control conditions, suggesting that PKA phosphorylation may play an important role in stabilizing CFTR at the plasma membrane (Fig. 6C,D). Compared to wild-type CFTR, the CS-induced internalization of CFTR^15SA^ was significantly reduced. Since CS-induced CFTR internalization was inhibited by forskolin or the removal of the R-domains’s serines, the phosphorylation state of CFTR was further investigated following CS exposure. Consistent with our previous observations, total CFTR decreased after lysis in mild detergent (1% NP40) with an increasing number of cigarettes (Fig. 6E,F)^16^. However, the amount of dephosphorylated plasma membrane CFTR increased after CS exposure. Together, these data indicate that the dephosphorylation of the R-domain promotes CFTR internalization. CFTR activity has been studied in humans using voltage-sensitive electrodes that can measure basal and agonist-induced CFTR activity. Indeed, based on these functional studies, CFTR is ~50% active *in vivo*, suggesting that it is to moderately phosphorylated in humans. That is, when measuring nasal PDs, there was a ~15 mV increase in PD when an apical low Cl^−^ solution was added, followed by another increase of ~15 mV when isoproterenol was added^58^. Thus, it is possible that CS could affect CFTR phosphorylation *in vivo*, but additional *in vivo* studies will be required before this mechanism of regulation can be better understood.

Protein phosphatases, including PP2A, have previously been associated with CFTR^59^. However, we found that okadaic acid, which inhibits PP2A (and PP1) had little effect on CS-induced CFTR internalzation (Fig. 7A,B). We have previously shown that CS induces lysosomal Ca^2+^ release and that CS-induced CFTR internalization is calcium-dependent^20^. Accordingly, we next tested whether a Ca^2+^-sensitive phosphatase was involved in CFTR internalization. Pretreatment with the calcineurin inhibitor cyclosporin A, prevented CS-induced CFTR trafficking (Fig. 7A,B) and CS increased cyclosporin-sensitive calcineurin activity (Fig. 7C,D). These data are consitent with a previous study which demonstrated that calcineurin is stimulated by lysosomal calcium release^60^. Moreover, the ability of CS to induce airway dehydration by internalizing CFTR in primary HBECs was prevented by cyclosporin A pretreatment, suggesting that the activation of calcineurin was functionally relevant. Thus, since CFTR internalization was forskolin/phosphorylation-sensitive post-CS (Figs 6, 7), we propose that CFTR dephosphorylation may be a novel physiological stimulus to initiate CFTR endocytosis and that this process is abberantly triggered by CS. Cyclosporin A has no effect on phagocytosis, a specialized form of endocytosis that occurs in macrophages^61^. However, in neurons, Cyclosporin A has been shown to inhibit Ca^2+^-dependent endocytosis^62^. Thus, whether the cyclosporin A-sensitivity is due to a general inhibition of endocytosis or to direct effects on CFTR remains to be determined.

Recently, the Forman-Kay lab have demonstrated that the Ca^2+^-sensitive kinase calmodulin interacts with CFTR’s R-domain, leading to CFTR phosphorylaton and activation^63^. In contrast, we have recently demonstrated that elevations in Ca^2+^ can inhibit CFTR via calcineurin^64^, which is consistent with our current data that CFTR is dephosphorylated and internalized. Thus, whether CFTR is activated or inhibted by CFTR may be dependent on the agonist that is used to elevate Ca^2+^ and which downstream kinases and/or phosphateases are subsequently activated. Importantly, we propose that phosphorylated CFTR has a lower rate of turnover and a longer residence time in the plasma membrane whilst dephosphorylated CFTR is less stable in the plasma membrane and more likely to internalize. We did not detect changes in other plasma membrane proteins after CS-exposure incluing Ano1, A2BR, P2Y2R and a GFP that was targeted to the cytosolic side of the plasma membrane (Fig. S1) and we have previously found no inhibitory effects of CS on ENaC^16^, suggesting that this is not non-specific. However, whether the effects of CS extend beyond CFTR to other membrane proteins, and/or can affect Ca^2+^-sensitive endoctosis remains to be determined.

Wong *et al*. recently demonstrated that acute CS extract exposure elevated cAMP and activated CFTR^65^. As these authors pointed out at the time, this activation is different to the CFTR inhibition seen by most other investigators. They attributed this to the difference in time course (i.e. they only observed CFTR activation after an acute exposure) and dose (i.e. they observed activation with lower doses of extract). In contrast, when we have exposed HBECs to CS, we have never observed an increase in ASL height, even though the recording period encompassed the period of CS exposure, and even though we were able to detect cAMP-dependent changes in ASL height in control cultures during this period^16^. Thus, since Wong *et al*. generated CS extract by bubbling smoke through warmed Ussing chamber solution, rather than by performing whole CS exposure, they may have been selecting for particular chemicals (e.g. the aqueous phase) of CS. Indeed, there are ~4,000 compounds in cigarette smoke and it is likely that many of these can interact with numerous proteins both inside cells and in the ASL by multiple mechanisms including adduct binding, redox interactions and altered cell signalling^66–68^. Further, the potential ROS-induced elevation in cAMP seen by Wong *et al*. would be predicted to phosphorylate CFTR, and be protective against CS-induced internalization, much in the fashion that we observed with forskolin (Fig. 6A,B). However, the ROS effect may be overwhelmed by higher levels of CS exposure seen in chronic smokers and observed with our whole smoke exposure system.

**Figure 8:**
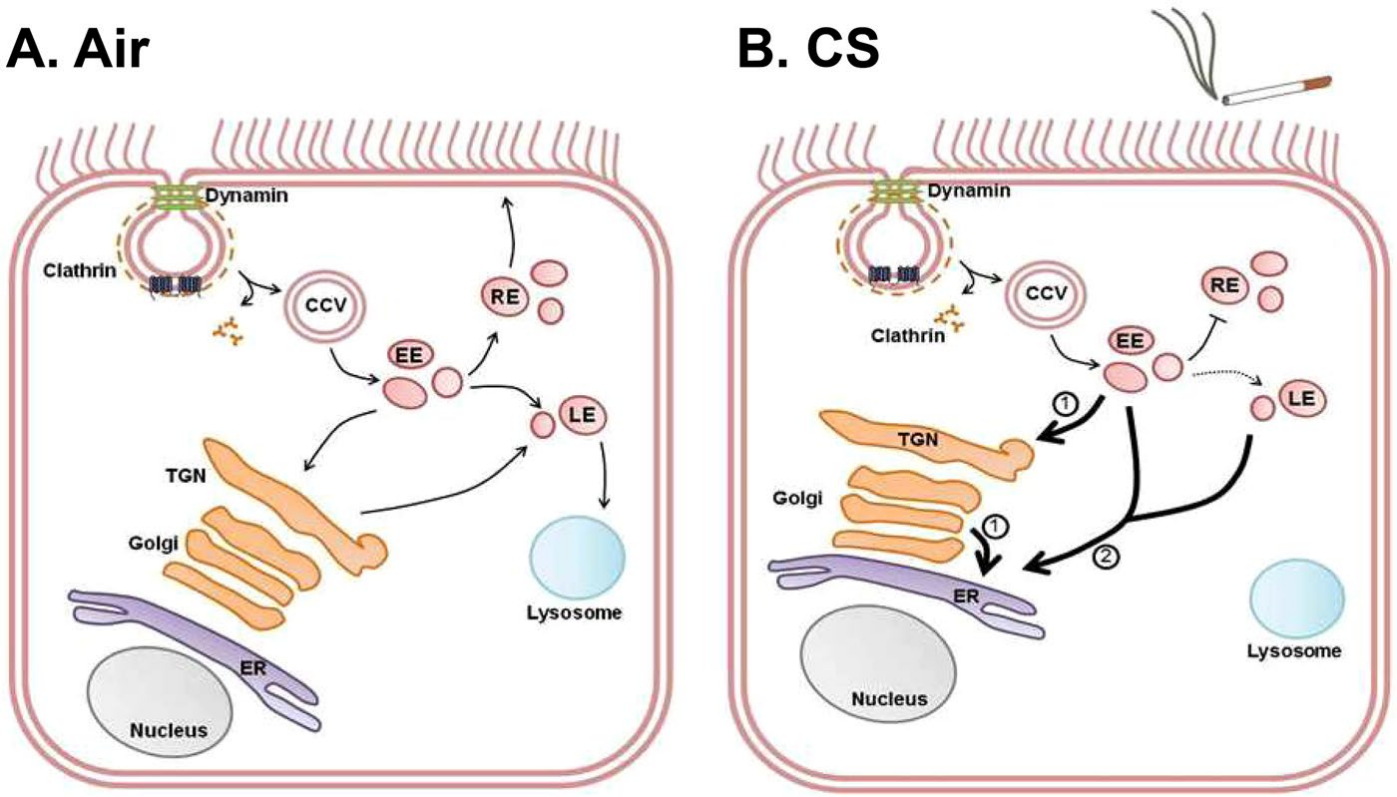
A proposed model for the endocytic pathway of CS-exposed CFTR. (**A**) In normal, air-exposed airway epithelia, CFTR endocytosis into clathrin-coated vesicles (CCV), which are cleaved off the plasma membrane by dynamin. CFTR is then trafficked to early endosomes (EE) and sorted to recycling endosomes (RE) and back to the plasma membrane or trafficking to the late endosomes (LE) and to the lysosome for degradation. (**B**) In CS-exposed epithelia, CFTR still enters CCVs in a dynamin-sensitive fashion and is trafficked to early endosomes. However, CFTR is no longer sent to recycling endosomes, or lysosomes. Instead, CFTR is trafficked to the ER. There are two proposed pathways for CS-induced retrograde trafficking of CFTR: (1) Direct ER-EE contacts allow the passage of CFTR to the ER. (2) CFTR from early and/or late endosomes is trafficked to the trans-Golgi-Network, through the Golgi and to the ER.

From an ion transport perspective, multiple groups, including ours, have demonstrated that CFTR is the only apical membrane channel affected by CS, whilst ENaC and Ano1 are surprisingly unperturbed by this exposure, both in HBECs and in humans^16,69^. We speculate that the observed effect on calcineurin (Fig. 7) likely affects membrane proteins beyond CFTR. However, more studies will be required to fully-understand calcineurin’s impact on other proteins. Moreover, the R-domain of CFTR is extremely flexible and in addition to being able to interact with other intracellular domains, such as the nucleotide binding domains, it also promiscuously interacts with many other proteins and thus may serve as the hub of the extensive CFTR interactome^70^. Thus, we hypothesize that CS-exposure and subsequent calcineurin-induced CFTR dephosphorylation of the R-domain alters CFTR’s interactome, leading to CFTR internalization and subsequent retrograde trafficking to the ER, as summarised in Fig. 8. Although complex, discerning the mechanism of internalization of CFTR by CS is of general interest in regards to both COPD and other CFTR-related diseases such as CF. Furthermore, the new finding that plasma membrane proteins accumulate in the ER may yield a better understanding of how CS affects cells in multiple organs of the body. Finally, our results also suggest that CFTR internalization after CS exposure should be studied in the context of an altered ER/unfolded protein response.

## Methods

All methods were carried out in accordance with UNC’s policies, guidelines and regulations.

### Solutions

Pyruvate Ringer’s solution (in mM): 120 NaCl, 12 NaHCO_3_, 24 HEPES, 1.2 MgCl_2_, 5.2 KCl, 1 NaPyruvate, 10 Glucose, 1.2 CaCl_2_, 0.25 EGTA, 0.1% Albumin (w/v), pH 7.4. Phosphate-buffered saline (PBS) (in mM): 2.7 KCl, 1.8 KH_2_PO_4_, 137 NaCl, 9.9 Na_2_HPO_4_, pH 7.4. In some cases, (PBS^++^) was supplemented with 1 mM MgCl_2_ and 1 mM CaCl_2_. Pierce’s lysis buffer: 25 mM Tris-HCl pH 7.4, 150 mM NaCl, 1 mM EDTA, 1% NP-40 (v/v) and 5% glycerol (v/v). Biotinylation lysis buffer: 0.4% sodium deoxychlorate (w/v), 50 mM EGTA, 10 mM Tris HCl, 1% NP40 (v/v) and 1X protease inhibitor. Borate buffer (in mM): 85 NaCl, 4 KCl, 15 NaB_4_O_2._ Tris-buffered saline with Tween 20 (TBST): 137 mM NaCl, 20 mM Tris, 0.1% Tween-20, pH 7.4.

### Cell culture

Human embryonic kidney (HEK) 293T cells were cultured in Dulbecco’s modified Eagle’s medium (DMEM) with 4.5 g/L glucose, supplemented with 100 Units/mL penicillin, 100 µg/mL streptomycin, and 10% foetal bovine serum (FBS, Sigma Aldrich, v/v). Baby Hamster Kidney (BHK) cells stably expressing wild type CFTR (BHK^CFTR^) or mutated CFTR lacking all 15 PKA phosphorylation sites (CFTR^15SA^) were cultured in DMEM:F12 medium supplemented by 100 Units/mL penicillin and 100 µg/mL streptomycin, 10% FBS (v/v) and 50 mg/mL methotrexate (Teva Pharmaceuticals). Human bronchial epithelial cultures (HBECs) were obtained from main stem and lumbar bronchi from human excess donor lungs. The protocols used were approved by the University of North Carolina Medical School Institutional Review Board (grandfathered under the CF Center Tissue Procurement and Cell Culture Core IRB protocols)^71^. Informed consent for tissue sample donation was obtained from all participants and/or their legal guardians. Primary HBECs were seeded on collagen coated 0.4 µm polyester membrane semi-permeable 12 mm culture inserts (Corning, transwell-clears) and maintained at air liquid interface for 3–4 weeks at 37 °C and 5% CO_2_ or plated onto glass coverslips and imaged 24 h later.

### Plasmids

Wild-type CFTR N-terminally labelled with GFP or RFP as indicated were gifts from Dr. Bruce Stanton at Dartmouth College (USA). Exotope/HA-CFTR was a gift from Drs. John Riordan and Martina Gentzsch at UNC-Chapel Hill (USA). Ano1-GFP was a gift from Dr. Criss Hartzell at Emory University (USA). Cathepsin B-mCherry was a gift from Dr. Bonnie Sloane (Wayne State University, USA). P2Y2-R-GFP was a gift from Dr H. Kendal Harden at UNC-Chapel Hill (USA) and A2BR-GFP was sub-cloned and generated in house. mRFP-clathrin light chain was a gift from Dr. Ari Helenius (Addgene plasmid # 14435). Rab5-DsRed; Rab11-DsRed and Rab7-DsRed were a gift from Dr. Sandra Schmid (Addgene plasmid # 34682 and # 34683, respectively); BHK cells stably expressing CFTR^15SA^ were kindly provided by Dr. Jack Riordan (UNC-Chapel Hill). BHK cells stably expressing wtCFTR were kindly provided by Dr. Martina Gentzsch (UNC-Chapel Hill). GFP- CFTR was subcloned into vector pcDNA3.1^72^. Site-directed mutagenesis was performed using the Quick Change Site Directed Mutagenesis Kit (Agilent Technologies). Primers were designed using Quick Change Primer Design program (Agilent Technologies) and purchased from Eurofins/MWGOperon. The plasmid was transformed into XL 10-Gold Ultracompetent cells (Agilent Technologies) according to manufacturer’s protocol. The plasmid was then amplified by mini-prep (Qiagen) and all mutant plasmids were verified by sequencing across the open reading frame before use. Premature stop codons were introduced to generate truncation mutations (GFP-CFTR^L1254X^ and GFP-CFTR^K1174X^).

### Cigarette smoke exposure

Kentucky 3R4F Reference Cigarettes were used in all smoke exposure experiments. An LM1 smoke machine (Borgwaldt) was used to perform all CS exposures and a Cambridge filter pad placed in the line to remove the autofluorescent particulate phase^20^. All cigarettes were smoked with a puff volume of 35 mL over a duration of 2 s. Approximately 13 puffs of CS were applied at a rate of 1 puff every 30 s.

### Immunocytochemistry

For all internalization assays, cells (HEK293T and BHK^CFTR^) were seeded at 75,000 cells per well on 25 mm glass coverslips in 6 well plates. HEK293T cells were transfected 24 h after seeding with 0.5–1 µg DNA according to manufacturer’s instructions. Experiments were performed 48 h after transfection. BHK^CFTR^ cells were used for experiments 48 h post-seeding. All cells were exposed to CS or air as described previously. After CS or air exposure, cells were incubated over time in 1 mL media at 37 °C before fixing in 4% paraformaldehyde for 5 min at room temperature or 100% methanol for 15 min at −20 °C. Following fixation, cultures were blocked at room temperature with agitation for 1 h in PBS with 10% (vol./ vol.) normal goat serum and 5% (vol./ vol.) bovine serum albumin. In some cases, wild-type or CFTR^15SA^ were labelled with monoclonal anti-CFTR 596 and 570 antibodies purchased from Cystic Fibrosis Foundation Therapeutics and kindly provided by Dr J. Riordan (UNC-Chapel Hill). Calreticulin was detected with anti-calreticulin polyclonal IgG antibody (Affinity BioReagents) and GM130 was detected with anti-GM130 D6B1 rabbit monoclonal IgG antibody (Cell Signalling Technology). Cells were washed and incubated with secondary antibody anti-rabbit labelled with Alexa Fluor 488, 568 or 633 or anti-mouse labelled with Alexa Fluor 488 (Life Technologies).

For surface labelling studies, HEK293T cells were seeded as described above on cover slips and transiently transfected with 0.5 µg HA-CFTR. After 24 h, cells were cooled to 4 °C in HEK293T media that also contained 1% BSA and 5% normal goat serum for 1 h. We then added mouse anti-HA conjugated to Alexa-488 in BSA/goat serum for 1 h. After 3 washes at 4 °C in ice-cold PBS, cells were placed in media, warmed up to 37 °C and exposed to air or CS as described above. Cultures were then fixed in 4% PFA for 30 min at room temperature and blocked for 1 h with PBS containing BSA and goat serum. Some cultures were fixed at 4 °C and not exposed to air or CS, as an additional naïve control. All cultures were then probed with the rabbit anti-calreticulin antibody (Affinity BioReagents), washed, stained with a goat anti-rabbit secondary antibody, stained with DAPI and then imaged on the SP8 confocal microscope.

Where indicated, HEK293T cells were transiently co-transfected with 0.5–1 µg wild-type GFP-CFTR alone, or GFP-CFTR and Ano1-mCherry, rab5-DsRed, rab7-DsRed, rab11A-DsRed and mRFP-clathrin light chain. All cultures were imaged on a Leica SP 5 or SP8 confocal microscope using a 63 × 1.40 or 100 × 1.49 numerical aperture plan apochromatic Leica oil objectives. To measure internalization, fluorescence was quantified using Image J software (NIH Freeware, http://rsb.info.nih.gov/ij/). In brief, images were opened up as 8 bit, grayscale stacks and regions of interest were drawn around portions of the plasma membrane and intracellularly (excluding the area that obviously contained the nucleus). 6 cells per coverslip were analysed, which included 6 plasma membrane and 6 intracellular regions. In all cases, mean fluorescence intensity was obtained. Since the background fluorescence was close to zero, no background subtraction occurred.

To determine the percentage co-localization, images were overlaid, and the mean Pearson’s correlation coefficient was determined using the LAS-AF software (Leica) in order to yield the percentage colocalization.: The following calculation was automatically used by the LAS AF software to determine the percentage colocalization rate between colocalized areas and background within the ROI. *Percent colocalization* = *colocalization area/area foreground*, *where area foreground* = *image area/area background*.

### Acceptor-photobleaching Förster resonance energy transfer

FRET was performed as described^20^. HEK293T cells co-transfected with 0.5 µg GFP-CFTR and 0.5 µg RFP-CFTR were treated with air or CS and fixed 48 h after transfection. Förster resonance energy transfer (FRET) experiments were performed using a Leica SP5 confocal microscope with a 63 × 1.30 NA plan apochromatic glycerol immersion objective. The donor (GFP-CFTR), was excited at 488 nm and the emission collected between 495 nm to 549 nm, and the acceptor (RFP-CFTR) was excited at 561 nm and emission collected between 580 nm to 654 nm. The FRET efficiency was measured using ImageJ by measuring a change in donor fluorophore fluorescence intensity after photobleaching of the acceptor fluorophore, using the following calculation: FRET efficiency (%E) = ((donor^p^°^stbleach^ − donor^prebleach^)/donor^postbleach^) × 100. All data presented as mean FRET efficiency (%E) ± (standard error of measurement).

### Cell surface biotinylation

Cultures were cooled to 4 °C and washed 3x in ice cold PBS^++^. The cultures were then agitated at 4 °C with 100 µg/µL biotin in borate buffer on the apical surface of the monolayer. FBS (10% vol./ vol.) was applied to the basolateral side and was maintained throughout incubation with biotin to ensure biotinylation of only the apical membrane. Excess biotins unable were quenched with 10% FBS. Cells were washed with ice cold PBS^++^ before lysis with 100 µL biotinylation lysis buffer at room temperature for 10 mins. The lysates were centrifuged for 5 min at 5000 × g to remove cell debris. Protein concentrations were calculated using the Bradford assay (Pierce) and samples were diluted in lysis buffer to ensure the same amount of protein was loaded in each tube. The lysates were rotated overnight with NeutrAvidin beads (ThermoFisher). The following day, the beads were washed 3 times with ice cold PBS and eluted with 10% 2-mercaptoethanol and 2x lithium dodecyl sulphate (LDS) buffer (Biorad; 40% glycerol (v/v), 4% lithium dodecyl sulfate, 4% Ficoll-400, 0.8 M triethanolamine-Cl pH 7.6, 0.025% phenol red, 0.025% Coomassie G250, 2 mM EDTA disodium). The membrane fractions were loaded on a gel and gel electrophoresis was performed at 150 mV for 1 h. The gels were then transferred, overnight at 4 °C to PDVF membranes. Membranes were blocked in 5% milk for 1 h at room temperature and probed for total CFTR with primary anti-CFTR 596 IgG2b and for dephosphorylated CFTR with primary anti-CFTR 217 IgG1 purchased from the Cystic Fibrosis Foundation Therapeutics and kindly provided by Dr J. Riordan (UNC). The membranes were washed a minimum of 3 × 10 min in TBST and probed with anti-mouse conjugated to horseradish peroxidase (Jackson ImmunoResearch). Blots were detected with Clarity enhanced chemiluminescence (ECL; Biorad) and visualised using a Chemidoc western blot imager (Biorad).

### Measurement of calcineurin phosphatase activity

Calcineurin activity was determined using a colorimetric assay as per manufacturer’s instructions (Enzo Life Sciences). HEK293T cells were seeded onto 60 mm culture dishes at a density of 10^6^ per dish and tested 24 h later. Following treatment, cells were washed twice with ice-cold Tris buffered saline solution (20 mM Tris, 150 mM NaCl, pH 7.2) and lysed in a solution containing (in mM); 50 Tris, 0.1 EDTA, 0.1 EGTA, 1 DTT, 0.2% NP-40, pH 7.5 with a protease inhibitor tablet and stored at −80 °C. Excess phosphates and nucleotides were removed from the lysates by passing the samples through a chromatography column and the desalted samples were stored at −80 °C. To ensure an equal amount of protein was run in the assay for each sample, a bicinchoninic acid (BCA) assay was run according manufacturer’s instructions. 3 µg of protein per sample was used for the calcineurin phosphatase assay. Total phosphatase activity in the samples was detected by addition of the phosphopeptide substrate, RII, in assay buffer. The assay plate was then equilibrated to the reaction temperature of 37 °C for 10 min and sample lysates were added to the assay plate at 37 °C for 30 min. The free phosphate was then measured by the addition of Biomol Green reagent and colour was allowed to develop for 30 min at 37 °C. Absorbance was measured at 620 nm and data were background corrected.

### Airway surface liquid height measurements

The airway surface liquid of primary well-differentiated HBECs was labelled with PBS containing tetramethylrhodamine-dextran (1 mg/mL). Perfluorocarbon (50 µL) was added to all cultures mucosally to prevent dehydration of the airway surface liquid during imaging as described^20^. XZ images were obtained at 20 predetermined points per culture using a Leica SP8 confocal microscope with an automated stage and a 63X glycerol immersion objective.

### Automated image acquisition

HEK293T cells were seeded at a density of 40,000 per well on 96 well plates. Cells were transfected using Lipofectamine 2000 (ThermoFisher Sci, Waltham, MA) following the manufacturer’s protocol. 24 h later, fluorescence was imaged using a Cytation 5 automated imaging system as described^73^. Before exposing cells to CS, media was replaced with 25 µl of FluoroBrite. Cells were then exposed to CS using a Borgwaldt LX-1 smoke machine coupled to a 3D-printed manifold that allowed for direct CS exposure to 96 well plates as described and reimaged 1 h later^73^.

### Image analysis and statistics

All quantification of images was performed using ImageJ (NIH Freeware, http://rsb.info.nih.gov/ij/) or LAS-AF (Leica). Graphs were produced using Prism 4.00 (GraphPad Software). All data are given as mean ± SEM unless stated otherwise and were checked for normal distribution. Where applicable, statistical significance was calculated using the Kruskal-Wallis test with Dunn’s multiple comparison post-test or two-way ANOVA with Dunn’s or Sidak’s multiple comparison post-test as appropriate. P values of ≤0.05 were considered significant.

## Supplementary information

Supporting information for: Cigarette Smoke Exposure Induces Retrograde Trafficking of CFTR to the Endoplasmic Reticulum
