# Supplementary material for: Cigarette Smoke Exposure Induces Retrograde Trafficking of CFTR to the Endoplasmic Reticulum
Source: Sci Rep. 2019 Sep 20;9:13655. doi: 10.1038/s41598-019-49544-9 (PMC6754399; doi:10.1038/s41598-019-49544-9)
Supplement: Supplementary file 1 — Supporting information for: Cigarette Smoke Exposure Induces Retrograde Trafficking of CFTR to the Endoplasmic Reticulum [file 41598_2019_49544_MOESM1_ESM.pdf]

Supporting information for:

Cigarette Smoke Exposure Induces Retrograde Trafficking of CFTR to the Endoplasmic Reticulum

Abigail J. Marklew[1, 2], Waseema Patel[1, 2], Patrick J. Moore[1], Chong D. Tan[1], Amanda J. Smith[1, 3], M. Flori Sassano[1], Michael A. Gray[2] and Robert Tarran[1, 3]

[1]Marsico Lung Institute, University of North Carolina, Chapel Hill, NC, USA; [2]Institute for Cell and Molecular Biosciences, Newcastle University, Newcastle-upon-Tyne, UK; [3]Department of Cell Biology & Physiology, University of North Carolina, Chapel Hill, NC, USA.

*Correspondence to: Robert Tarran
7102 Marsico Hall, 125 Mason Farm Road,
University of North Carolina at Chapel Hill,
Chapel Hill, NC, 27599.
Email: robert_tarran@med.unc.edu
Contact: 919-966-7052

Running Title: Cigarette Smoke and CFTR Trafficking

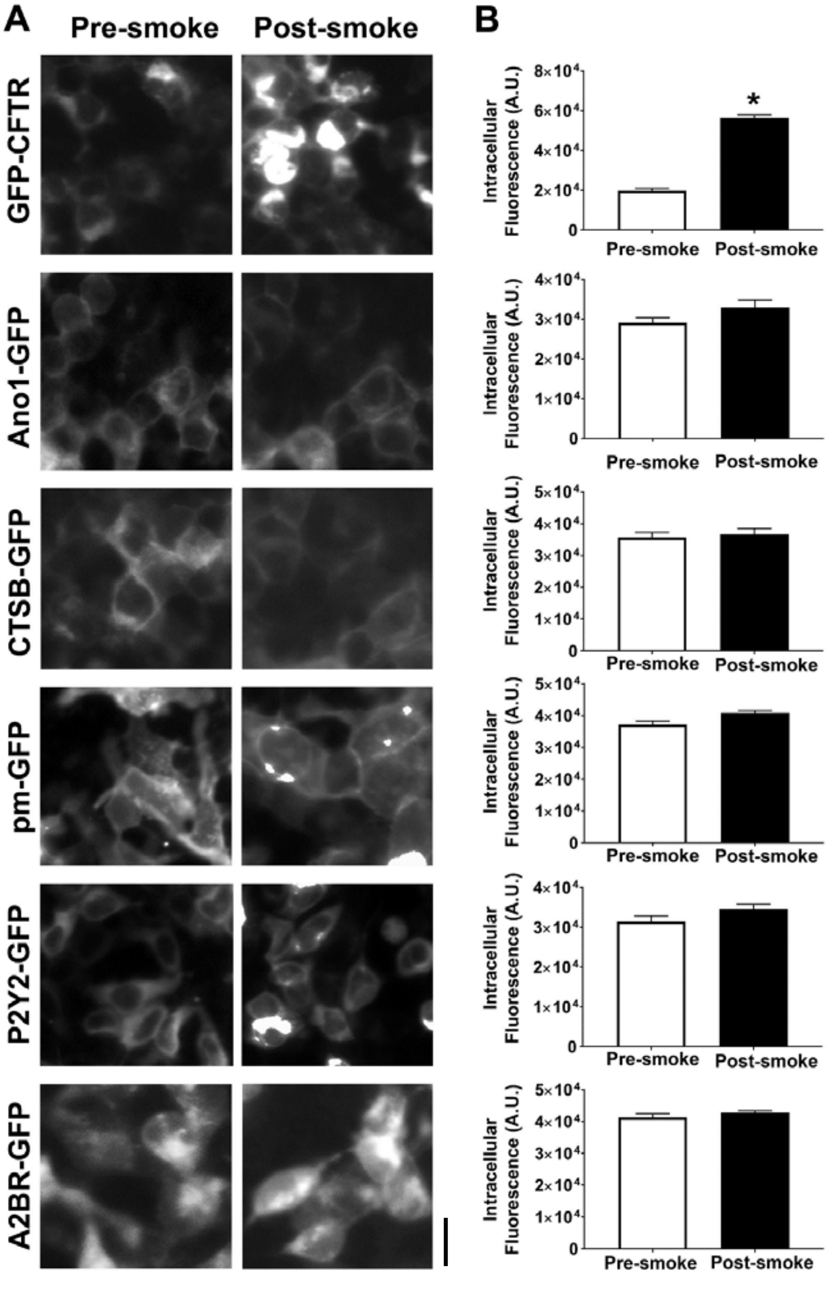

Figure S1. CFTR but not other membrane proteins are internalized by CS exposure. HEK293T cells were cultured in 96 well plates, trasnfected as indicated and 24 h later, exposed to CS using a 3D-printed manifold and imaged by automated microscopy 1 h later. **(A)** Representative epifluorescent images of GFP- or YFP- labeled constructs ± CS exposure. **(B)** Mean intracellular fluorescence pre/post CS exposures as indicated. * = $p < 0.05$ different to pre-CS exposure. N=10 per condition. Scale bar is 10 µm.

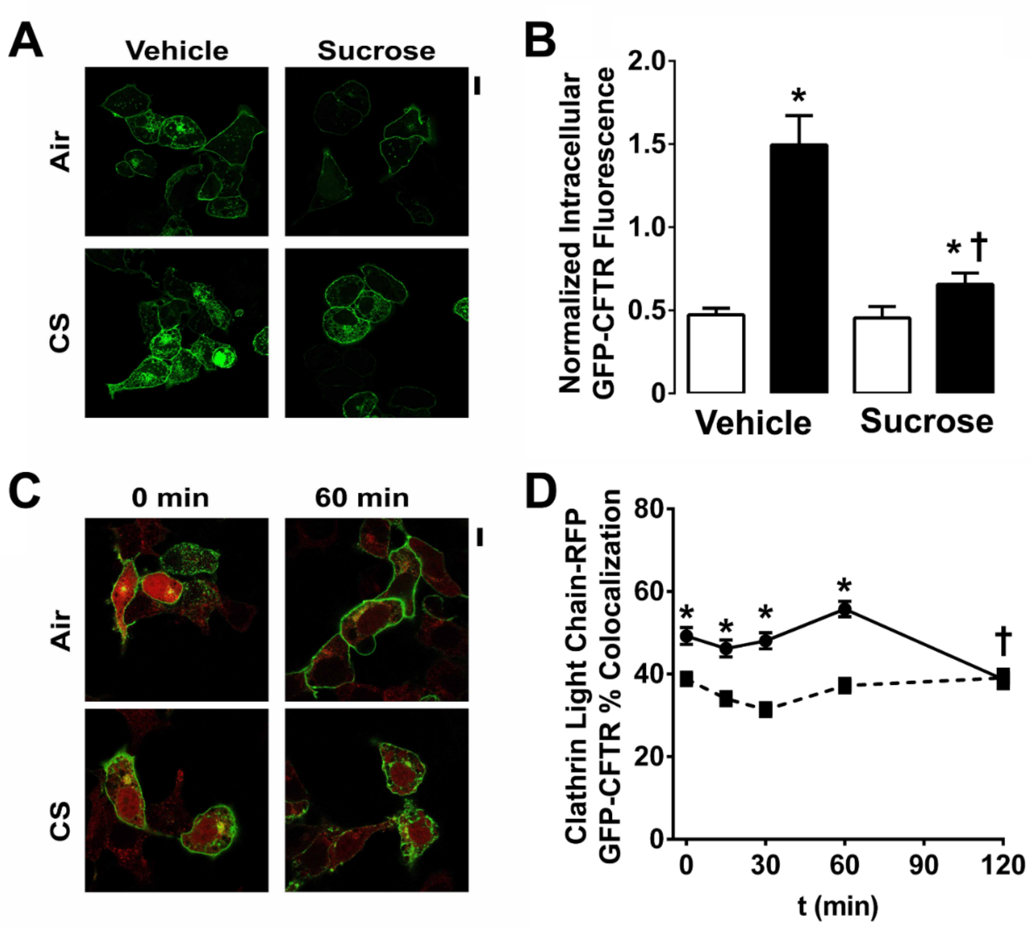

Figure S2. CS-induced internalization of CFTR is clathrin-dependent. **(A)** Representative confocal images of GFP-CFTR ± 0.45 M hypertonic sucrose in air and CS exposed HEK293T cells. **(B)** Mean intracellular fluorescence in air- (open bars) or CS- (closed bars) exposed cells treated with vehicle or 0.45 M sucrose. N = 91 - 182 cells per bar from 3 independent experiments. **(C)** Confocal micrographs of HEK293T cells transfected with GFP-CFTR (green) and clathrin light chain-mRFP (red) exposed to air or CS and incubated over time. **(D)** Mean percentage colocalization between CFTR and clathrin over time. ■, air exposure; ●, CS exposure. N = 49 - 85 cells per time point from 3 separate experiments. * = $p < 0.01$ different to air. † = $p < 0.01$ different to CS control/CS t=0. Scale bars are 10 μm.

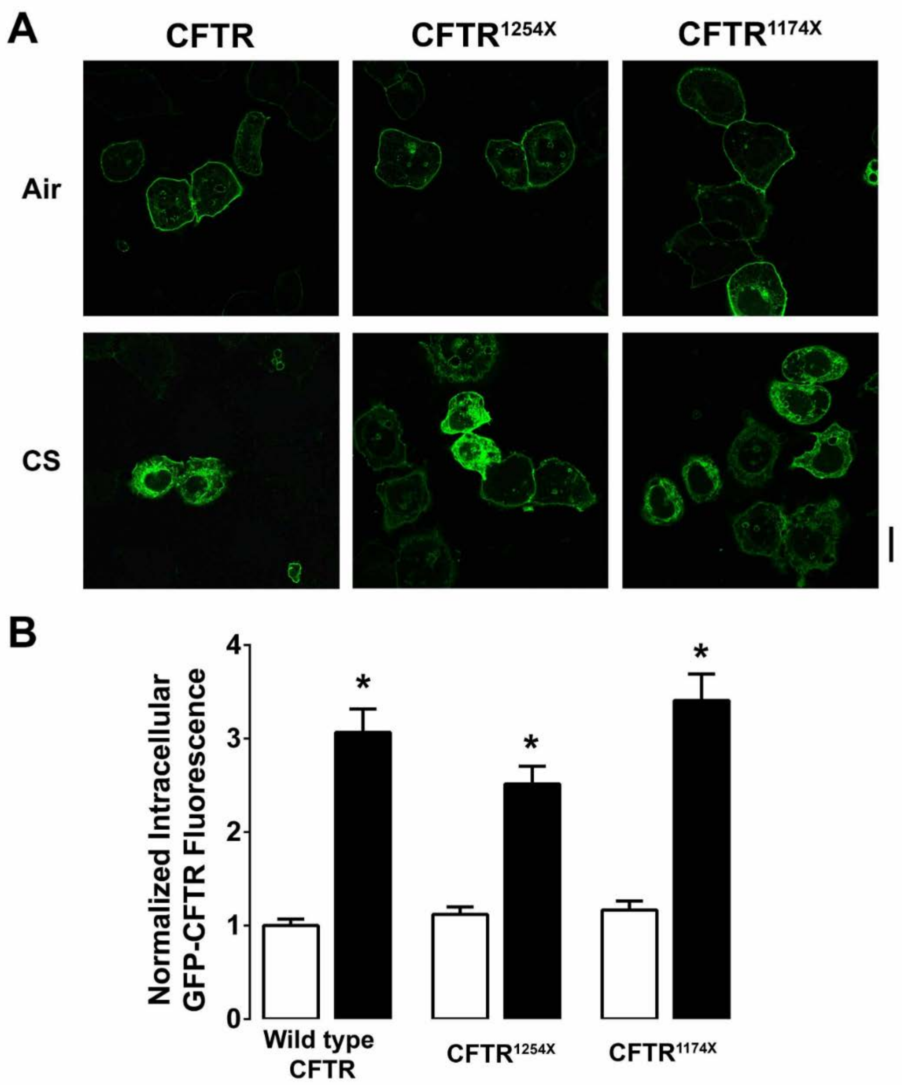

Figure S3. The C-terminus and NBD2 of CFTR are not required for CS-induced CFTR internalization. (A) Representative confocal images of HEK293T cells expressing GFP-CFTR, GFP-CFTR^{K1174X} or GFP-CFTR^{L1254X} after air and CS exposure. **(B)** Mean intracellular fluorescence in air (open bars) or CS (closed bars) exposed HEK293T cells trasfected with wild-type or mutant CFTR. N = 103 - 213 cells from three independent experiments. * = $p < 0.01$ different to air controls. Scale bar is 10 µm.

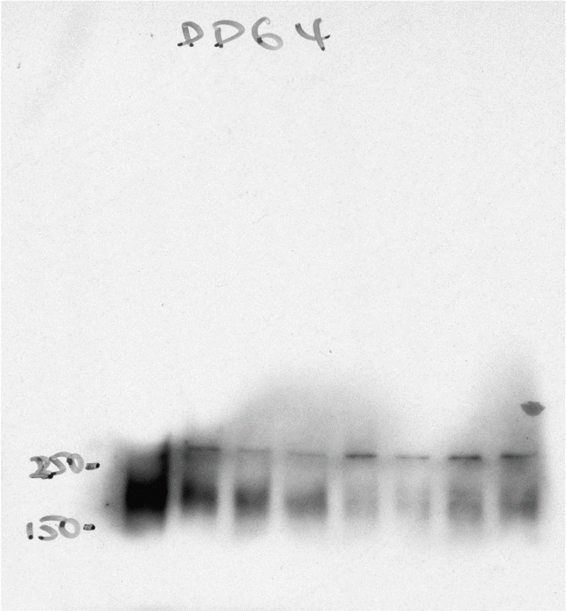

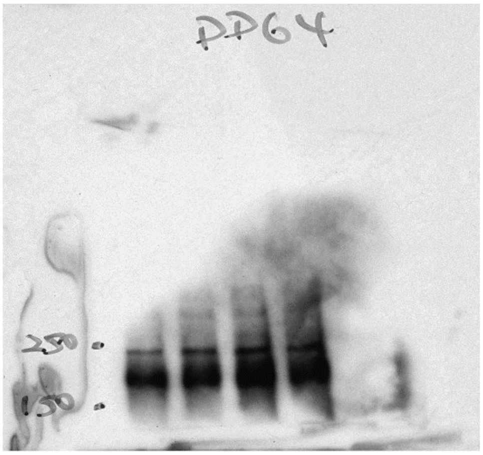

CFTR Antibody #217 CFTR Antibody #596

Figure S4. Full-length gels used for Figure 6 (CS-induced CFTR dephosphorylation is required for CFTR internalization). The representative data are from one HBEC donor and were reprobed using two different CFTR antibodies. The two lanes on the left of each gel were used for Figure 6.
